# The comparison of clinical and biological characteristics between *IDH1* and *IDH2* mutations in gliomas

**DOI:** 10.1186/s13046-016-0362-7

**Published:** 2016-05-31

**Authors:** Hao-Yuan Wang, Kai Tang, Ting-Yu Liang, Wei-Zhong Zhang, Ji-Ye Li, Wen Wang, Hui-Min Hu, Ming-Yang Li, Hui-Qing Wang, Xiao-Zheng He, Zhi-Yuan Zhu, Yan-Wei Liu, Shi-Zhong Zhang

**Affiliations:** Department of Neurosurgery, Zhujiang Hospital, Southern Medical University, 253# Gongye Road, Guangzhou, China; Department of Neurosurgery, Beijing Tiantan Hospital, Capital Medical University, No. 6 TiantanXili, Dongcheng District, Beijing, 100050 China; The National Key Clinical Specialty. The Engineering Technology Research Center of Education Ministry of China Guangdong Provincial Key Laboratory on Brain Function Repair and Regeneration Department of Neurosurgery, Zhujiang Hospital, Southern Medical University, Guangzhou, China; Beijing Neurosurgical Institute, Capital Medical University, Beijing, China; Chinese Glioma Cooperative Group (CGCG), Beijing, China; Center for Brain Disorders Research, Capital Medical University, Beijing, 100069 China

**Keywords:** Glioma, IDH mutation, Whole transcriptome sequencing, DNA methylation analyzes

## Abstract

**Background:**

Mutations in isocitrate dehydrogenase 1 (*IDH1*) and isocitrate dehydrogenase 2 (*IDH2*) are frequent in low-grade gliomas and secondary glioblastomas (sGBM). Because they yield the same oncometabolite, D-2-hydroxyglutarate, they are often treated as equivalent and pooled. The objective of this study was to provide insight into the differences between *IDH1* and *IDH2* mutant gliomas.

**Methods:**

To investigate the different clinical and molecular characterization between *IDH1* mutant and *IDH2* mutant gliomas, we studied 811 patients with *IDH1* mutations, *IDH2* mutations and *IDH1/2* wild-type. In addition, whole-transcriptome sequencing and DNA methylation data were used to assess the distribution of genetic changes in *IDH1* and *IDH2* mutant gliomas in a Chinese population-based cohort.

**Results:**

Among 811 gliomas in our cohort, 448 cases (55.2 %) harbored an *IDH1* mutation, 18 cases (2.2 %) harbored an *IDH2* mutation and 345 cases (42.6 %) harbored an *IDH1/2* wild-type. We found that *IDH1* and *IDH2* are mutually exclusive in gliomas, and *IDH2* mutations are mutually exclusive with *PTEN*, *P53* and *ATRX* mutations. Patients with *IDH2* mutations had a higher frequency of *1p/19q* co-deletion (*p* < 0.05) than *IDH1* mutant patients. In addition, a Gene Set Enrichment Analysis (GSEA) showed that *IDH2* mutant gliomas were associated with the oxidative phosphorylation gene set, and the four most representative biological processes for genes commonly altered by hypermethylation in *IDH2* mutant gliomas were the regulation of cell proliferation, cell motion, cell migration and response to hypoxia. Patients with *IDH2* mutant gliomas exhibited longer Overall survival (OS) (*p* < 0.05) and longer Progression-free survival (PFS) (*p* < 0.05) than patients with *IDH1/2* wild-type gliomas. However, their OS and PFS did not differ from that of *IDH1* mutant patients.

**Conclusions:**

Our study revealed an intrinsic distinction between *IDH1* and *IDH2* mutant gliomas, and these mutations should be considered separately because their differences could have implications for the diagnosis and treatment of *IDH1/2* mutant gliomas.

## Background

Isocitrate dehydrogenase (*IDH*) enzymes encode the NADP^+^-dependent isocitrate dehydrogenase, which catalyzes the oxidative decarboxylation of isocitrate to form an α-ketoglutarate (α-KG). *IDH1* and *IDH2* proteins share a high degree of sequence similarity (70 % in humans) and are encoded by distinct genes (*IDH1*, 2q33 and *IDH2*, 15q26). Mutations in *IDH1* and *IDH2*, which represent the most frequently mutated metabolic genes in human cancer, are implicated to be mutated in more than 50–80 % of low-grade gliomas and secondary glioblastomas (sGBM), 10 % of intrahepatic cholangiocarcinoma, 20 % of acute myeloid leukemia (AML), 56 % of chondrosarcomas, and over 10 % of melanoma cases [1–5]. Although *IDH1* and *IDH2* are highly similar and catalyze identical reactions, *IDH1* is localized in the cytosol and *IDH2* is found in the mitochondrial matrix. In addition, the spectrum of cancers and their subtypes are different. For example, *IDH1* mutations are predominant in gliomas, chondrosarcoma, and cholangiocarcinoma, whereas *IDH1* mutations and *IDH2* mutations are equally common in AML. Despite their different physiological characteristics, most genomic studies of the molecular landscapes in human cancer have frequently combined *IDH1* mutations and *IDH2* mutations as a single functional group.

Glioma, the most common primary brain tumor, is classified as grade I to IV based on histopathological and clinical criteria established by the 2007 World Health Organization (WHO) [6]. WHO grade I gliomas are often curable by surgical resection, whereas WHO grade II or III gliomas are invasive and have a poor prognosis. WHO grade IV tumors (glioblastomas), the most invasive tumors, feature a median survival of only 16 months, even after aggressive treatment consisting of surgery, radiation therapy, and chemotherapy [7]. In 2008, the genes encoding *IDH1* were found to be mutated in low-grade gliomas and a subset of sGBM [8]. In subsequent studies, *IDH1* mutations were reported to occur in 70–80 % of WHO grade II or III astrocytomas, oligodendrogliomas, and oligoastrocytomas, whereas a small group (3–5 %) were found to harbor *IDH2* mutations [1]. This pattern contrasts that observed in AML, which features similar rates of *IDH1* (6.6 %) and *IDH2* mutations (10.8 %) [9]. Moreover, mutations of *IDH1* and *IDH2* are mutually exclusive in gliomas, and biochemical investigations showed that *IDH1* and *IDH2* mutations differ in D-2-hydroxyglutarate (D-2HG) production in gliomas [10]. This difference suggests that *IDH1* and *IDH2* mutations may impact different cellular pathways and exert different tumorigenic effects. To investigate the different clinical and molecular characterization between *IDH1* mutant and *IDH2* mutant gliomas, we studied a cohort of 811 patients consisting 448 *IDH1* mutant, 18 *IDH2* mutant and 345 *IDH1/2* wild-type gliomas. We performed whole-transcriptome sequencing and DNA methylation analyses of the samples obtained from patients. We compared the mutational landscapes of *IDH1* and *IDH2* mutant gliomas, their clinical associations, overall survival, and progression-free survival. Our aim was to provide insight into the differences between *IDH1* and *IDH2* mutant gliomas.

## Methods

### Patients and tumor samples

Glioma samples were obtained from 811 patients with gliomas, including 448 *IDH1* mutant, 18 *IDH2* mutant and 345 *IDH1/2* wild-type gliomas, which were composed of 577 low grade (II + III) gliomas, including 193 diffuse astrocytoma, 39 anaplastic astrocytomas, 49 low-grade oligodendrogliomas, 27 anaplastic oligodendrogliomas, 186 oligoastrocytomas, 83 anaplastic oligoastrocyotmas and 234 glioblastomas. These patients underwent surgery and were followed-up at Beijing Tiantan hosipital from 2004 to 2014. Clinicopathologic data, including gender, age, pathologic diagnosis and the results of molecular analysis were obtained. When the cases were classified as secondary GBMs based on biopsy-proven preexisting low-grade gliomas, 29 cases (12.4 %) were secondary GBM and the remainder were primary GBM (205 cases, 87.6 %).

Whole transcriptome sequencing of 161 gliomas and DNA methylation profile of 44 glioma samples, were obtained from Chinese Glioma Genome Atlas (CGGA) database (http://www.cgga.org.cn) [11–13]. All these samples were histologically graded according to 2007 WHO classification of tumours of the nervous systems [6]. Written informed consent was obtained from all donors. Clinical investigations were performed after approval by the local research ethics committee and in accordance with the ethical principles.

### IDH mutation

Genomic DNA was isolated from frozen tissues with a QIAamp DNA Mini Kit (Qiagen) according to the manufacturer’s protocol. The DNA concentration and quality were evaluated with a Nano-Drop ND-1000 spectrophotometer (NanoDrop Technologies, Houston, TX). The pyrosequencing of *IDH1/2* mutations was supported by Gene-tech (Shanghai, China) and performed on a Pyro-Mark Q96 ID System (Qiagen, Valencia, Calif). The primers 5′-GCTTGTGAGTGGATGGGTAAAAC-3′, 5′-Biotin-TTGCCAACATGACTTACTTGATC-3′, for *IDH1* and 5′-ATCCTGGGGGGGACTGTCTT-3′, 5′-Biotin-CTCTCCACCCTGGCCTACCT-3′ for *IDH2* were used for PCR amplification, and the primers 5′-TGGATGGGTAAAACCT-3′ for *IDH1* and 5′-AGCCCATCACCATTG-3′ for *IDH2* were used for pyrosequencing [13].

### Gene set enrichment analysis

To identify the gene sets related to particular biological processes present in IDH-mutant patients, gene expression profiling and a gene set enrichment analysis (GSEA) were performed as described previously [14].

### Statistical analysis

Survival distributions were estimated with a Kaplan-Meier survival analysis, and the log-rank test was used to assess the significance of differences between stratified survival groups using the GraphPad Prism 5.0 statistical software. The differences among patients in baseline clinical and molecular features according to *IDH1* and *IDH2* mutational status were tested using the Fisher’s exact and Wilcoxon rank sum tests for categoric and continuous variables, respectively. Gens that were differently methylated between IDH2 mutant and IDH1 mutant tumors were obtained using the standard two-sampled *t*-test with unequal variance and sample size. To adjust for multiple comparisons, we applied the Benjamini-Hochberg method to control the False Discovery Rate at 5 %. We further filtered the list of significant genes by retaining those which exhibited at least 1.5-fold difference in gene expression between IDH2 mutant and IDH1 mutant in our final comparisons. Student’s *t*-test was performed using SPSS 16.0. A two-sided *p* value < 0.05 was considered significant.

## Results

### Clinical and molecular characterization of *IDH2* mutations

Among a total of 811 gliomas, *IDH2* mutations were identified in 18 cases (2.2 %) (Table 1). *IDH2* mutations were found in 0.5 % of pGBM (1/215), 3.4 % of sGBM (1/29) and 2.8 % (16/577) of low grade gliomas, while *IDH1* mutations are found in 14.1 % (29/205) of pGBM, 55.2 % (16/29) of sGBM and 69.8 % (403/577) of low grade gliomas. Combined *IDH1* and *IDH2* mutations were found in 14.6 % (30/205) of pGBM, 58.6 % (17/29) of sGBM and 72.6 % (419/577) of low grade gliomas.

**Table 1:** The summary of the materials analysed in this study

Pathological diagnosis	WHO grade	*n* = 811	*IDH1* mutation No. (%)	*IDH2* mutation No. (%)	IDH mutation total No. (%)
Diffuse astrocytoma	II	193	138 (71.5)	2 (1.0)	140 (72.5)
Anaplastic astrocytoma	III	39	14 (35.9)	0 (0)	14 (35.9)
Oligodendroglioma	II	49	38 (77.6)	3 (6.1)	41 (83.7)
Anaplastic oligodendroglioma	III	27	19 (70.4)	1 (3.7)	20 (74.1)
Oligoastrocytoma	II	186	147 (79.0)	9 (4.8)	156 (83.8)
Anaplastic oligoastrocytoma	III	83	47 (56.6)	1 (1.2)	48 (57.8)
Subtotal (grades II and III)		577	403 (69.8)	16 (2.8)	419 (72.6)
Primary GBM	IV	205	29 (14.1)	1 (0.5)	30 (14.6)
Secondary GBM	IV	29	16 (55.2)	1 (3.4)	17 (58.6)
Subtotal (Glioblastoma)	IV	234	45 (19.2)	2 (0.9)	47 (20.1)
Total		811	448 (55.2)	18 (2.2)	466 (57.4)

**Fig. 1:**
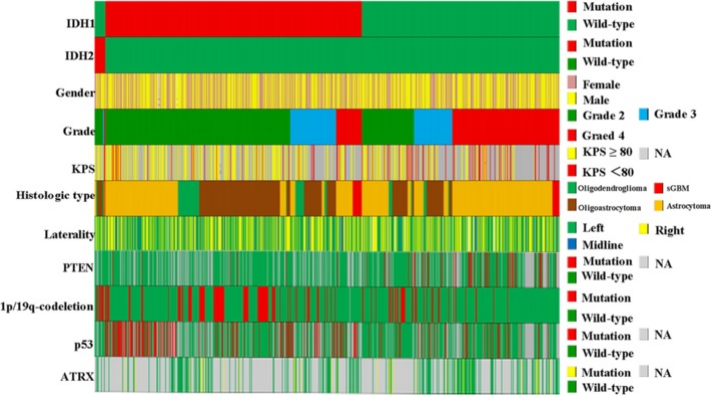
Overview of clinical and molecular characterization of the cohort (*n* = 811). Each column represents a patient

As shown in Fig. 1 and Table 2, patients with mutations in *IDH2* did not differ from *IDH1*-mutant patients in terms of age, gender, WHO grade, KPS, histologic type and laterality (Table 2). To characterize the molecular features of *IDH2* mutant gliomas, we analyzed associations between *IDH2* mutations and other mutational events. Patients with *IDH2* mutations had a higher frequency of *1p/19q* co-deletion (*p* < 0.05) and a lower frequency of *P53* mutation (*p* < 0.05) than *IDH1* mutant patients (Table 3). Strikingly, the presence of *IDH2* mutations and *PTEN* mutations, *P53* mutation and *ATRX* mutation did not correlate (Fig. 1 and Table 3).

**Table 2:** Clinical characteristics according to *IDH* mutational status in gliomas

Clinical characteristic		*N* = 811	*IDH1*-Mutated	*IDH2*-Mutated	*IDH1*/*IDH2*-Wild-type	*P* (*IDH1*-Mutated VS *IDH1*/*IDH2*- Wild-type)	*P* (*IDH2*-Mutated VS *IDH1*/*IDH2*- Wild-type)	*P* (*IDH1*-Mutated VS *IDH2*-Mutated)
			No. (%)	No. (%)	No. (%)			
Age, years	<45	423	285 (67.4)	13 (3.1)	125 (29.5)	<0.001	<0.05	0.456
	≥45	388	163 (42.0)	5 (1.3)	220 (56.7)			
Median age, years			39.1	37.3	45.0	<0.001	<0.05	0.456
Range			17.0–66.0	26.0–56.0	9.0–81.0			
Gender	Male	495	265 (53.5)	11 (2.2)	219 (44.2)	0.113	0.801	0.824
	Female	316	186 (58.9)	7 (2.2)	123 (38.9)			
WHO Grade	II + III	577	403 (69.8)	16 (2.8)	158 (27.4)	<0.001	<0.001	0.883
	IV	234	45 (19.2)	2 (0.9)	187 (79.9)			
KPS score	<80	74	26 (35.1)	2 (2.7)	46 (62.2)	<0.001	0.376	0.514
	≥80	253	173 (68.4)	7 (2.8)	73 (28.8)			
	NA	483	248	9	226			
Histologic type	Oligodendroglioma	76	57 (75.0)	4 (5.3)	15 (19.7)	<0.001	<0.001	0.218
	Oligoastrocytoma	269	194 (72.1)	10 (3.7)	65 (24.2)			
	Astrocytoma	437	181 (41.4)	3 (0.7)	253 (57.9)			
	sGBM	29	16 (55.2)	1 (3.4)	12 (41.4)			
Laterality	Left	380	211 (55.5)	9 (2.4)	160 (42.1)	<0.05	0.891	0.985
	Right	366	203 (55.5)	8 (2.2)	155 (42.3)			
	Midline	53	33 (62.3)	1 (1.9)	19 (35.8)			
	NA	12	1	0	11

**Table 3:** Molecular characteristics according to *IDH* mutation in glioma

Molecular Characteristic		*IDH1*-Mutated	*IDH2*-Mutated	*IDH1*/*IDH2*-WT	P (*IDH1*-Mutated VS *IDH1*/*IDH2*-WT)	P (*IDH2*-Mutated VS *IDH1*/*IDH2*-WT)	P (*IDH1*-Mutated VS *IDH2*-Mutated)
*PTEN*	Mutation	6	0	43	<0.001	0.098	0.605
	Wild-type	314	14	218			
	NA	128	4	84			
*1p/19q* Co-deletion	Absent	341	9	307	<0.001	<0.001	<0.05
	Present	106	9	34			
	NA	1	0	1			
*P53*	Mutation	215	0	232	<0.001	0.154	<0.05
	Wild-type	115	14	34			
	NA	118	4	79			
*ATRX*	Mutation	13	0	15	0.084	0.638	0.728
	Wild-type	107	5	102			
	NA	328	13	228			

### Gene set enrichment analysis for *IDH2* mutant patients

**Fig. 2:**
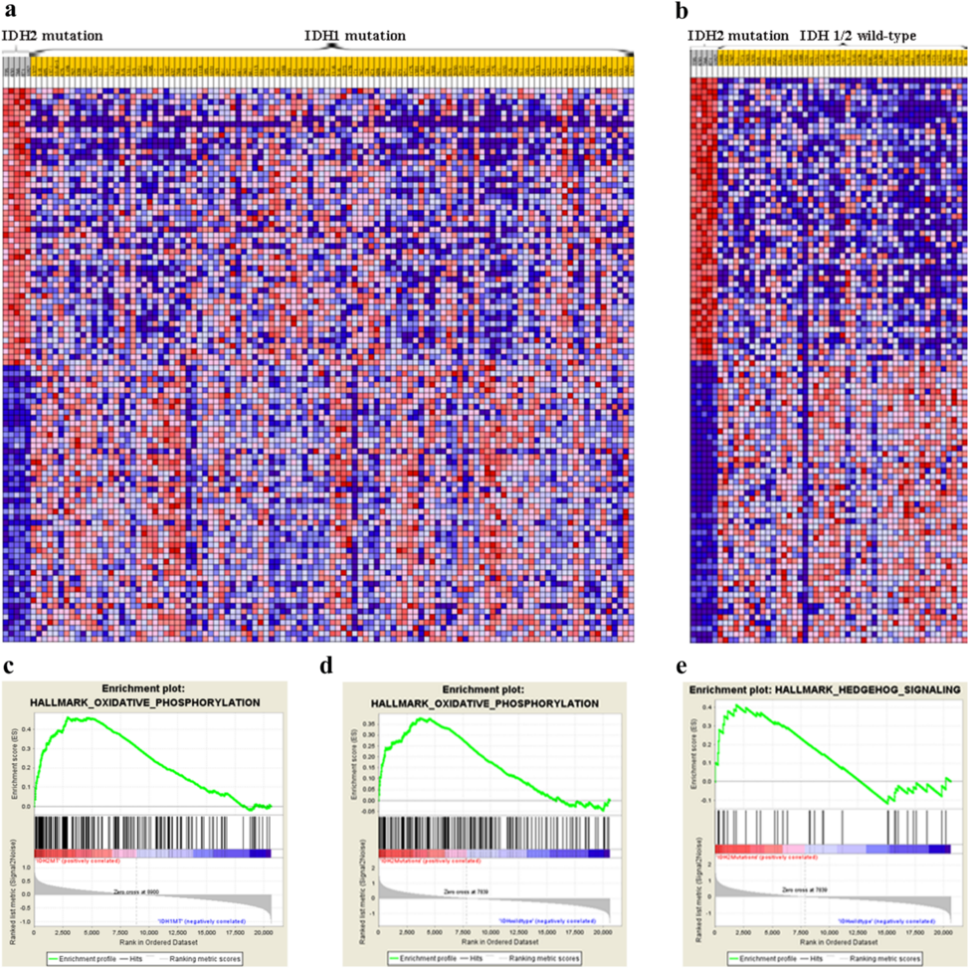
Gene Set Enrichment Analysis (GSEA) of overexpressed genes in glioma harboring *IDH2* mutations. Each row represents a gene, and each column indicates a glioma with an *IDH2* mutation, *IDH1* mutation or *IDH1/2* wild-type. Red indicates upregulated genes, and blue indicates downregulated genes. **a** Expression levels of genes annotated in *IDH2* mutant gliomas compared to *IDH1* mutant gliomas. **b** Expression levels of genes annotated in *IDH2* mutant gliomas compared to *IDH1/2* wild-type gliomas. **c** one representative plot of GSEA from a. **d**-**e** two representative plots of GSEA from (**b**)

To gain biologic insight into the potentially significance of *IDH2* mutations, we compared the whole-transcriptome sequencing expression profiles of 5 *IDH2* mutant patients with 109 *IDH1* mutant patients and 47 *IDH1/2* wild-type patients. First, we used a Gene Set Enrichment Analysis (GSEA) to compare the global gene expression profiles of the *IDH2* mutant and *IDH1* mutant gliomas. The result showed that the oxidative phosphorylation gene set was upregulated (FDR q-value = 0; Fig. 2a/c). We then compared the whole-transcriptome sequencing expression profiles of the *IDH2* mutant and *IDH1/2* wild-type gliomas (Fig. 2b). The results showed that the oxidative phosphorylation gene set (FDR q-value < 0.001; Fig. 2d) and hedgehog signaling set were upregulated (FDR q-value < 0.05; Fig. 2e).

### *IDH2* mutant gliomas exhibit DNA methylation profiles distinct from those of *IDH1* mutant gliomas

**Fig. 3:**
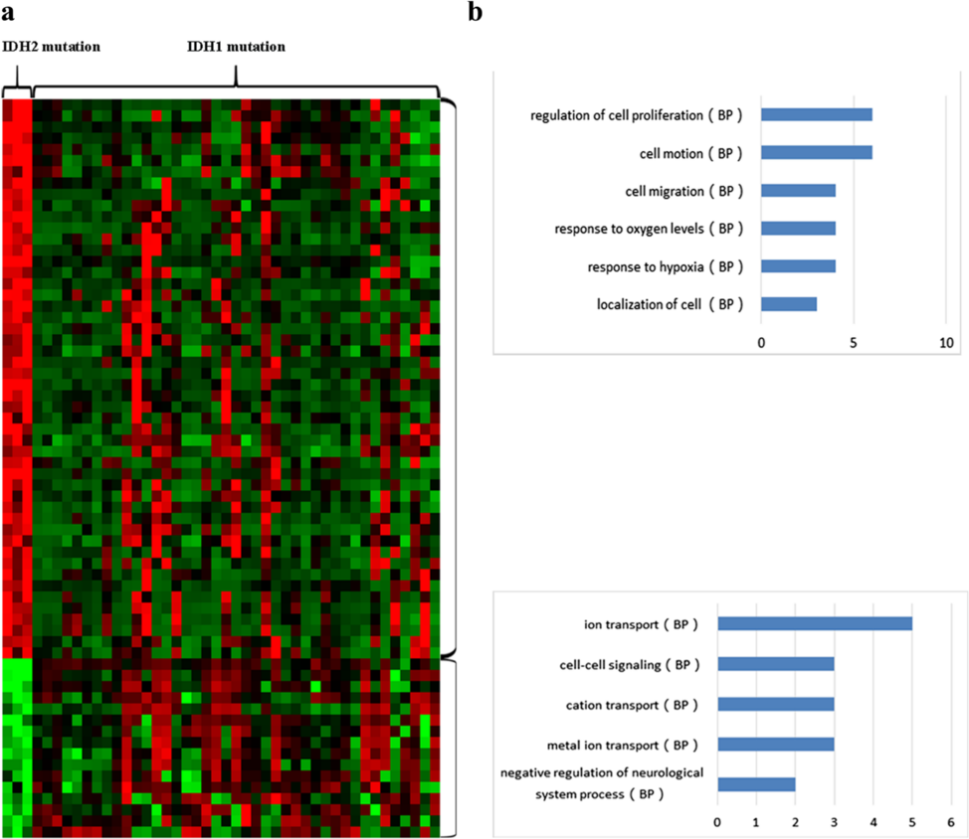
Clustering analysis of DNA methylation in *IDH2* mutant gliomas. **a** The pattern of DNA methylation was associated with *IDH2* mutant gliomas using a one-dimensional hierarchical clustering analysis. **b** Functional enrichment analysis of associated genes, indicating the functional roles of gene sets in different subgroups. Enrichment results for biological processes were obtained from the GO database. The orders of biological processes listed in the histogram are based on the number of targets annotated in the biological process (BP)

Given the DNA methylation profiles of 3 *IDH2* mutant gliomas and 41 *IDH1* mutant gliomas, we used standard t-tests to identify differentially methylated regions. The methylation patterns of genes that correlated with *IDH2* mutant gliomas are shown in Fig. 3a using a one-dimensional hierarchical clustering analysis. The four most representative biological processes for genes commonly altered by hypermethylation were the regulation of cell proliferation, cell motion, cell migration and response to hypoxia (Fig. 3b). According to the hypomethylated genes, the three most representative biological processes were ion transport, cell-cell signaling, and cation transport (Fig. 3b).

### Associations of *IDH2* mutations with clinical outcome

**Fig. 4:**
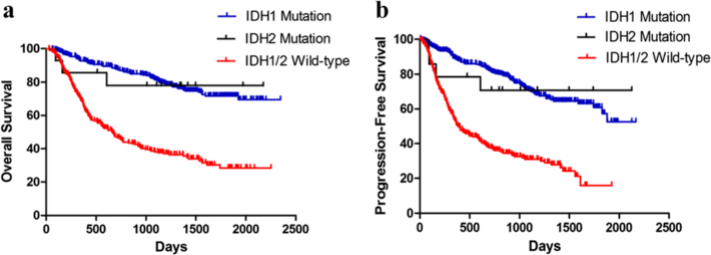
The Kaplan–Meier estimates for Overall survival (OS) (**a**) and Progression-free survival (PFS) (**b**) indicates that *IDH2* mutant gliomas associated with longer overall survival (*p* = 0.011) and longer progression-free survival (*p* = 0.011) than *IDH1/2* wild-type gliomas. However, the OS and PFS did not significantly differ between *IDH1* mutant and *IDH2* mutant gliomas

In our cohort, the presence of an *IDH2* mutation was associated with a longer overall survival (*p* < 0.05) and longer progression-free survival (*p* < 0.05) (Fig. 4a/b) than the presence of the *IDH1/2* wild-type gene. However, when considering all patients with *IDH2* mutations, the overall survival and time to recurrence did not differ from those of *IDH1* mutant patients (Fig. 4a/b). This result illustrates that the effects of *IDH2* mutation and *IDH1* mutation on clinical prognosis were similar.

## Discussion

Mutations in the *IDH1* and *IDH2* genes have been found in patients with gliomas and were initially identified in low-grade gliomas and secondary glioblastomas [1]. Strikingly, mutations in *IDH1* and *IDH2* are mutually exclusive in gliomas. Although the genetic and epigenetic landscapes of *IDH1* mutation gliomas have been extensively studied, whether *IDH2* mutation gliomas have unique genetic and epigenetic characteristics that can be used as targets for future intervention is unknown. In this report, we compared the clinical and molecular characteristics of glioma patients harboring *IDH1* and *IDH2* mutations.

Like mutations in *IDH1*, mutations in *IDH2* affect a conserved arginine residue (R172) in the substrate-binding site of the *IDH2* enzyme. In our cohort, the presence of an *IDH2* mutation did not correlate with the presence of *PTEN*, *P53*, and *ATRX* mutations, but a highly significant positive correlation was observed with the presence of a *1p/19q* co-deletion: 44.4 % of *IDH2* mutation patients harbored a *1p/19q* co-deletion. In malignant glioma, *IDH1* mutations are ubiquitous in tumor cells, and *IDH1* mutations precede secondary and tertiary lesions, suggesting that *IDH1* mutations are an early causative event in the genesis of gliomas [15–17]. A pathology study of multiple biopsies from the same patient found that *IDH1* mutations occurred before the acquisition of *P53* mutations and *1p/19q* loss of heterozygosity (LOH) [16], suggesting that *IDH1* mutations may result in cellular stress that leads to the mutation of *P53* and *1p/19q* loss. However, *IDH2* mutations and *PTEN*, *P53* and *ATRX* mutations were mutually exclusive, suggesting that the microenvironment of *IDH2* mutations may not create cellular stress that leads to the other mutations, which needs further research to fully elucidate.

Tumor cells often take up nutrients in excess of their bioenergetic needs and shunt metabolites into pathways that support tumor progression [18–20]. During cell proliferation, tumor cells depend on aerobic glycolysis to meet their bioenergy needs and generate intermediates for macromolecule biosynthesis. One study demonstrated that glioma cells harboring mutant *IDH1* may maintain cell proliferation via the glutamate metabolism pathway [21]. In our study, GSEA was performed for *IDH2* and *IDH1* mutations, yielding enriched gene sets related to oxidative phosphorylation, which is critical to tricarboxylic acid (TCA) cycle, in the *IDH2* mutation subset. This finding corroborates that of a previous study [22, 23]. *IDH2* is localized in the mitochondria and participates in the TCA to produce energy, whereas *IDH1* is localized in the cytoplasm and peroxisomes [24]. Consequently, does energy production in *IDH2*-mutated gliomas favor oxidative phosphorylation over aerobic glycolysis? These interesting findings should be verified in more cases before accepting them as general characteristics of *IDH2*-mutated gliomas. Future work should focus on the potential of therapeutically targeting compensatory metabolic pathways in *IDH2*-mutant gliomas.

## Conclusion

In conclusion, our results describe the clinical and biological characteristics of *IDH1* and *IDH2* mutations in gliomas. Understanding the underlying biology of the differences in outcome observed for *IDH1* and *IDH2* mutant gliomas will be important for future studies and may lead to the development of novel approaches to therapy.

## Abbreviations

CGGA: Chinese Glioma Genome Atlas
GSEA: gene set enrichment analysis
IDH1: isocitrate dehydrogenase 1
IDH2: isocitrate dehydrogenase 2
OS: overall survival
PFS: progression-free survival
sGBM: secondary glioblastomas
